# The glucocorticoid receptor in recipient cells keeps cytokine secretion in acute graft-versus-host disease at bay

**DOI:** 10.18632/oncotarget.24602

**Published:** 2018-03-02

**Authors:** Tina Baake, Katharina Jörß, Jennifer Suennemann, Laura Roßmann, Hanibal Bohnenberger, Jan P. Tuckermann, Holger M. Reichardt, Henrike J. Fischer, Sybille D. Reichardt

**Affiliations:** ^1^ Institute for Cellular and Molecular Immunology, University Medical Center, Georg-August-University Göttingen, Göttingen, Germany; ^2^ Institute of Pathology, University Medical Center, Georg-August-University Göttingen, Göttingen, Germany; ^3^ Institute of Comparative Molecular Endocrinology, University of Ulm, Ulm, Germany; ^4^ Present address: Institute for Multiple Sclerosis Research and Neuroimmunology, University Medical Center, Georg-August-University Göttingen, Göttingen, Germany

**Keywords:** glucocorticoid receptor, myeloid cells, GvHD, IL-6, cytokine storm

## Abstract

Graft-versus-host disease (GvHD) is a life-threatening complication of hematopoietic stem cell transplantation (HSCT), which is caused by allogeneic T cells recognizing molecules of the recipient as foreign. Endogenous glucocorticoids (GC) released from the adrenal gland are crucial in regulating such inflammatory diseases. Here we demonstrate that genetically engineered mice, that are largely unresponsive to GC, suffer from aggravated clinical symptoms and increased mortality after HSCT, effects that could be tempered by neutralization of IL-6. Interestingly, selective ablation of the GC receptor (GR) in recipient myeloid cells resulted in fulminant disease as well. While histopathological analysis of the jejunum failed to reveal any differences between sick mice of both genotypes, systemic IL-6 and TNFα secretion was strongly increased in transplanted mice lacking the GR in myeloid cells briefly before the majority of them succumbed to the disease. Collectively, our findings reveal an important role of the GR in recipient cells in limiting the cytokine storm caused by GvHD induction.

## INTRODUCTION

GvHD is a severe inflammatory disease responsible for affliction and mortality in a majority of patients undergoing allogeneic HSCT, a treatment indicated for perilous hematological disorders involving the transfer of hematopoietic stem cells from a healthy donor into a preconditioned patient [1, 2]. In the first phase of GvHD, local inflammation caused by the conditioning regimen leads to the release of pro-inflammatory cytokines, which activate recipient antigen presenting cells (APC) [3]. Damage to the intestinal epithelium further results in the translocation of pathogen-associated molecules, thereby initiating an innate immune response [4]. In the second phase, T cells in the graft recognize molecules of the recipient as foreign, mostly unmatched MHC class I and II molecules but also polymorphic proteins, and subsequently start to proliferate and differentiate into effector cells [5]. In the final phase of the pathogenic cascade, cytokines and cytotoxic molecules are released by T cells and myeloid cells, which results in tissue damage in the intestine, liver and skin [1]. This process is generally observed within 100 days post transplant, characterized by symptoms such as erythroderma, diarrhea and jaundice, and referred to as acute GvHD (aGvHD). In contrast, chronic GvHD usually starts later and represents a distinct disease entity that involves systemic fibrosis and autoantibody production [6].

Despite considerable research efforts, treatment options for aGvHD remain unsatisfactory and reliable prognostic factors are scarce [7, 8]. As of yet, systemic GC administration is the only proven first-line therapy for aGvHD. If disease fails to ameliorate after treatment or in the case that GC are not tolerated, second-line therapy can be accomplished by using monoclonal antibodies that target key pathogenic molecules expressed by T cells such as CD25, CD52 or TNFα [8]. Since none of these agents is sufficiently powerful, continuation of GC treatment is recommended even after onset of second-line therapy. GC therefore remain essential in the management of aGvHD despite the complications that accompany their clinical use, including poor response rates and different adverse effects [9, 10]. Hence a better understanding of the mode of GC action in aGvHD is important.

Recipient myeloid cells play crucial roles in aGvHD. On the one hand allogeneic immune responses can be initiated by different host APC, and on the other hand mediators are released by macrophages of the host that contribute to tissue damage in target organs [3]. It is thus conceivable that the immunomodulatory activity of GC also involves effects on recipient macrophages, which can be polarized into distinct phenotypes [11]. Following classical activation, they assume a pro-inflammatory phenotype characterized by TNFα, IL-1β and IL-6 release and increased expression of costimulatory and MHC molecules [12]. In contrast, macrophages exposed to GC assume an anti-inflammatory phenotype characterized by markedly reduced cytokine secretion, diminished antigen presentation and increased phagocytosis. GC achieve these effects mostly by modulating gene expression, which either requires binding of the GR to regulatory genomic elements or its interaction with transcription factors such as NF-κB [13]. Eventually, GC hereby manage to keep inflammation in check, dampen T cell responses and initiate tissue repair, all of which contributes to their beneficial effects in the control of aGvHD.

Genetic mouse models have considerably helped us to understand the pathophysiological role of the GR in inflammatory diseases and their treatment. Ablating the GR in individual cell types such as myeloid cells (GR^lysM^) [14] or ubiquitously disrupting its dimerization interface (GR^dim^) [15] allowed to define the cellular targets of GC and their mode of action in mouse models of contact dermatitis, multiple sclerosis, sepsis, rheumatoid arthritis, acute lung injury, and allergic asthma [14, 16–20]. Animal models have also proven to be valuable in the analysis of aGvHD [21]. Experiments in mice for instance showed that adhesion molecules and chemokines in the intestinal tract are crucial targets of GC, and provided evidence that the functionality of CMV-specific T cells in aGvHD can be retained by selectively inactivating the GR [22, 23]. Studies from our group further revealed that the GR in transplanted T cells is essential to control disease severity in two mouse models of aGvHD, in particular by repressing the activity of CD8^+^ cytotoxic T cells [24]. In this study we tested the role of the GR in radioresistant cells of HSCT recipients, in particular macrophages, and found that endogenous GC keep cytokine secretion during aGvHD at bay. This finding suggests that selectively targeting GC to myeloid cells might offer a new strategy to interfere with aGvHD in affected patients.

## RESULTS

### Proper control of aGvHD requires an intact GR in recipient cells

**Figure 1:**
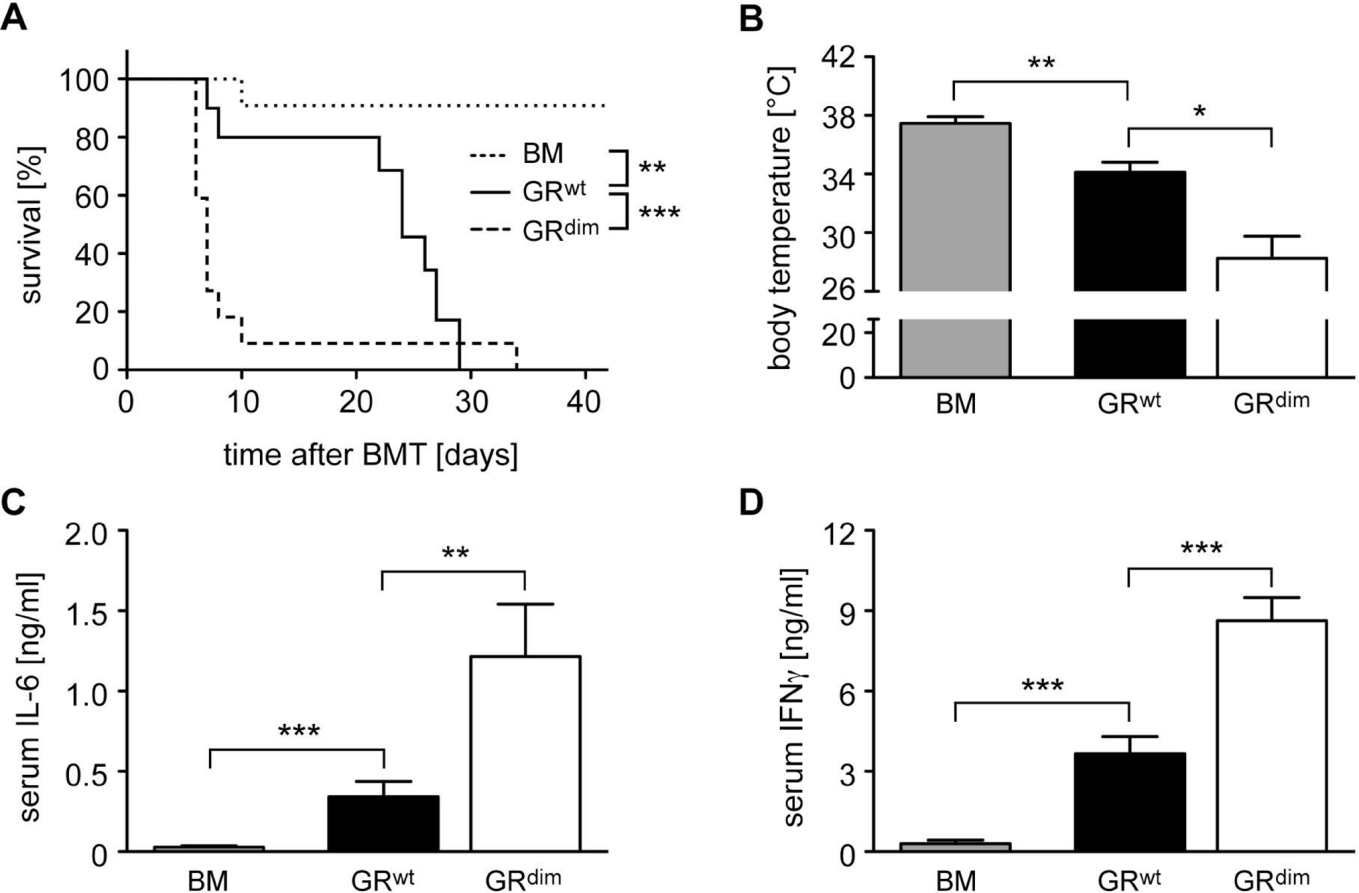
Mortality and clinical features of aGvHD in the GR^dim^ model GR^wt^ and GR^dim^ BALB/c mice were transplanted with BM and purified T cells from C57BL/6 wildtype mice; transfer of BM cells only served as a control. (**A**) Survival of mice was monitored for 42 days. *N* = 11 (BM), *N* = 10 (GR^wt^), *N* = 22 (GR^dim^); data pooled from multiple experiments. (**B**) Body temperature of mice was analyzed on day 6. *N* = 6 (BM), *N* = 7 (GR^wt^), *N* = 9 (GR^dim^); data pooled from multiple experiments. (**C**, **D**) Mice were sacrificed on day 6 and serum levels of IL-6 (C) and IFNγ (D) were analyzed by ELISA. *N* = 7 (BM), *N* = 9 (GR^wt^), *N* = 6 (GR^dim^) for IL-6; *N* = 7 (BM), *N* = 13 (GR^wt^), *N* = 15 (GR^dim^) for IFNγ; data pooled from multiple experiments. All values are depicted as the mean ± SEM. Survival curves were compared using the Gehan-Breslow-Wilcoxon test, statistical analyses of body temperature and cytokine levels were performed by Mann-Whitney *U* test (^*^p < 0.05; ^**^p < 0.01; ^***^p < 0.001; n.s.: non-significant).

We previously found that the GR in transplanted T cells was required to prevent fulminant aGvHD after allogeneic HSCT in mice [24]. Here we set out to explore the importance of the GR in recipient cells. Since mice with an ubiquitous deletion of the GR are not viable [25], we used GR^dim^ mice which express a GR with a defective dimerization interface [15] and are largely unresponsive to GC [26]. When we transferred bone marrow (BM) and mature T cells from wildtype C57BL/6 mice into irradiated GR^wt^ Balb/c recipients, mice died between day 10 and 30 post transplant (Figure 1A). In contrast, HSCT into GR^dim^ mice resulted in exacerbated aGvHD, and the majority of animals died within the first 10 days (Figure 1A). The body temperature of mice with aGvHD was reduced on day 6, briefly before the peak of the disease [24, 27], and this effect was more pronounced in transplanted GR^dim^ than GR^wt^ mice (Figure 1B). Furthermore, IL-6 and IFNγ serum levels on day 6 were significantly more increased in GR^dim^ than GR^wt^ mice, which is in line with the aggravated clinical symptoms of mutant mice (Figure 1C, 1D). We conclude that an intact GR in recipient cells is required to prevent fulminant disease and early lethality after allogeneic HSCT.

### Systemic IL-6 release impacts the severity of aGvHD

**Figure 2:**
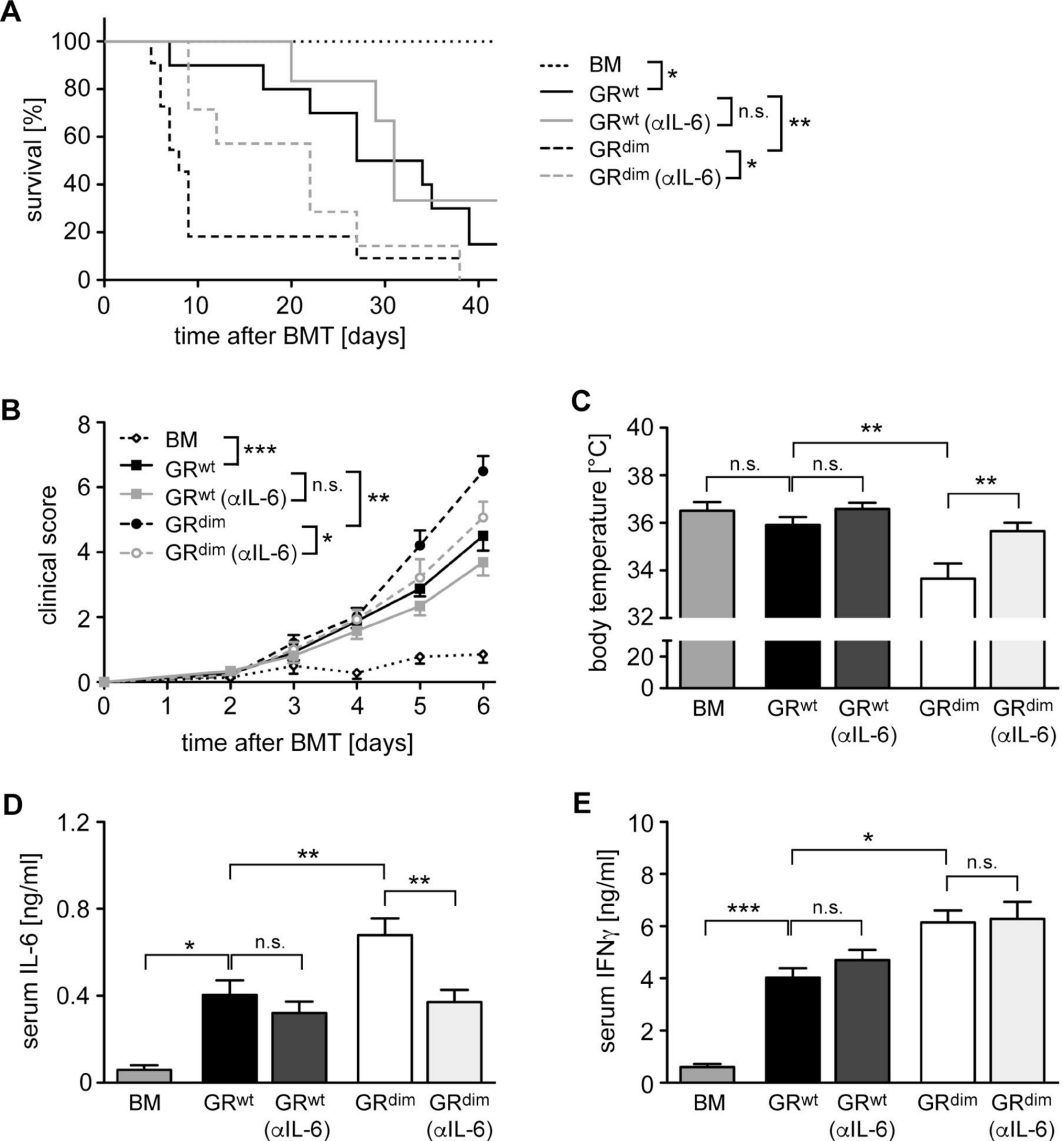
Impact of anti-IL-6 antibody treatment on mortality and clinical features of aGvHD in the GR^dim^ model GR^wt^ and GR^dim^ BALB/c mice were transplanted with BM and purified T cells from C57BL/6 wildtype mice. Some mice received an anti-IL-6 (αIL-6) antibody i.v. on day 2 (all panels) and day 6 (panel A); transfer of BM cells only served as a control. (**A**) Survival of mice was monitored for 42 days. *N* = 7 (BM), *N* = 10/6 (GR^wt^ ± αIL-6), *N* = 11/7 (GR^dim^ ± αIL-6); data pooled from multiple experiments. (**B**) Clinical scores of mice during the first 6 days after aGvHD induction (dead mice were considered with a score of 10). *N* = 7 (BM), *N* = 12/13 (GR^wt^ ± αIL-6), *N* = 19/14 (GR^dim^ ± αIL-6); data pooled from multiple experiments. (**C**) Body temperature of mice was analyzed on day 6. *N* = 7 (BM), *N* = 15/13 (GR^wt^ ± αIL-6), *N* = 22/13 (GR^dim^ ± αIL-6); data pooled from multiple experiments. (**D**, **E**) Mice were sacrificed on day 6 and serum levels of IL-6 (D) and IFNγ (E) were analyzed by ELISA. *N* = 7 (BM), *N* = 8/11 (GR^wt^ ± αIL-6), *N* = 14/12 (GR^dim^ ± αIL-6) for IL-6; *N* = 7 (BM), *N* = 8/12 (GR^wt^ ± αIL-6), *N* = 16/12 (GR^dim^ ± αIL-6) for IFNγ; data pooled from multiple experiments. All values are depicted as the mean ± SEM. Survival curves were compared using the Gehan-Breslow-Wilcoxon test, statistical analyses of body temperature and cytokine levels were performed by One-way ANOVA followed by Newman-Keuls multiple comparison test (^*^p < 0.05; ^**^p < 0.01; ^***^p < 0.001; n.s.: non-significant).

As previous studies provided evidence for an involvement of IL-6 in aGvHD [28, 29], we tested whether the elevated systemic secretion of this cytokine in GR^dim^ mice might be responsible for the devastating disease course in the mutants. To this end, GR^wt^ and GR^dim^ mice were treated with a neutralizing anti-IL-6 antibody on day 2 and 6 post transplant followed by monitoring the survival over 42 days. Another cohort of mice was sacrificed on day 6 after only one injection with the antibody and used for analysis. It turned out that the anti-IL-6 antibody therapy of GR^dim^ mice prevented early lethality, significantly reduced clinical scores and interfered with the drop in body temperature (Figure 2A–2C). The enhanced systemic IL-6 secretion in GR^dim^ mice as compared to GR^wt^ mice was no longer observed after antibody therapy as expected, whereas IFNγ levels in the mutants remained high (Figure 2D, 2E). It is noteworthy that anti-IL-6 treatment of GR^wt^ mice had only a marginal effect on mortality, disease severity and cytokine levels, which failed statistical significance (Figure 2A–2E). In summary, our data suggest that impaired GR function in recipient cells aggravates aGvHD due to an increased systemic release of cytokines such as IL-6.

### GR ablation in recipient myeloid cells exacerbates aGvHD

**Figure 3:**
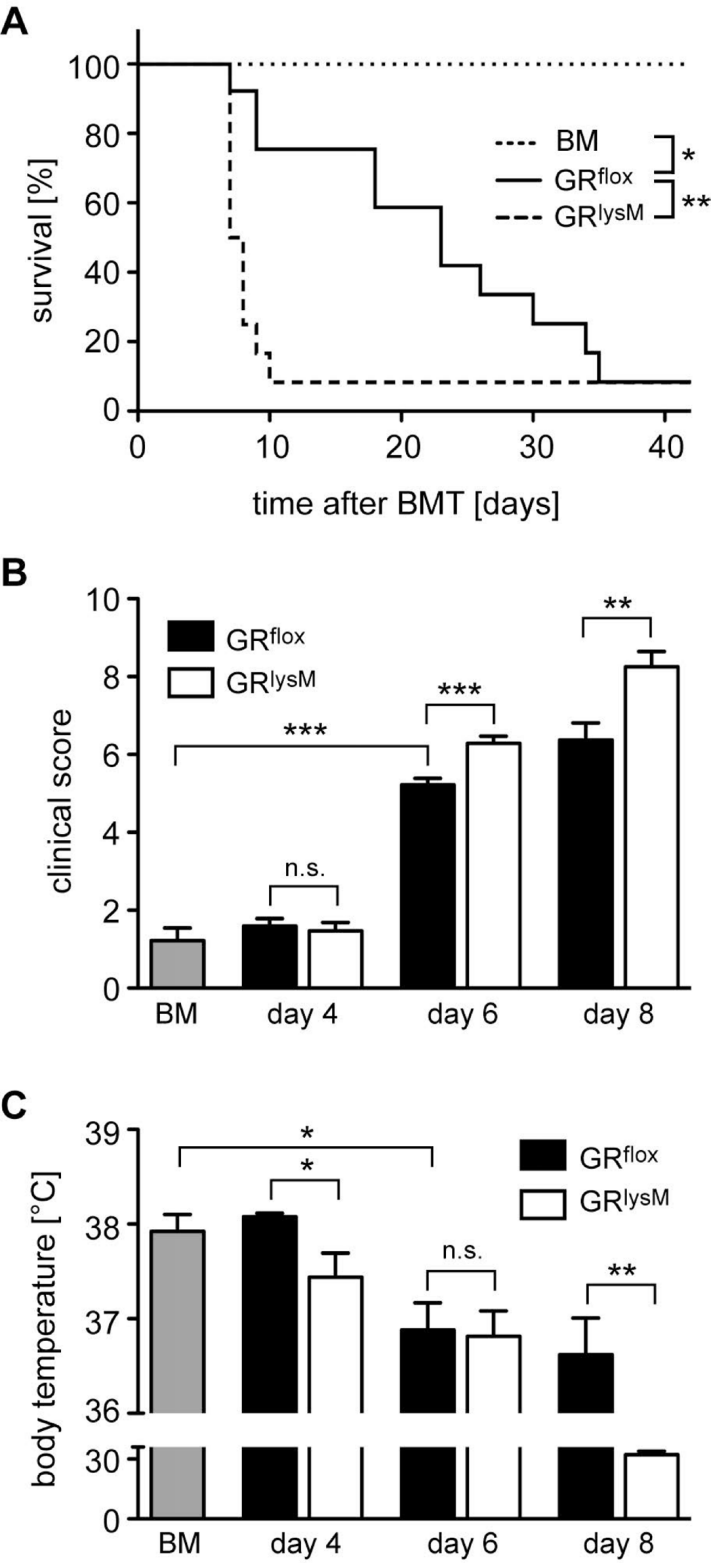
Mortality and clinical features of aGvHD in the GR^lysM^ model GR^flox^ and GR^lysM^ BALB/c mice were transplanted with BM and purified T cells from C57BL/6 wildtype mice; transfer of BM cells only served as a control. (**A**) Survival of mice was monitored for 42 days. *N* = 6 (BM), *N* = 14 (GR^flox^), *N* = 13 (GR^lysM^); data pooled from multiple experiments. (**B**) Clinical scores were determined on day 4, 6 and 8 after aGvHD induction (dead mice were considered with a score of 10); analysis of BM controls was performed on day 6. *N* = 9 (BM), *N* = 31/31 (GR^flox^/GR^lysM^; day 4), *N* = 39/40 (GR^flox^/GR^lysM^; day 6), *N* = 19/20 (GR^flox^/GR^lysM^; day 8); data pooled from multiple experiments. (**C**) Body temperature was determined on day 4, 6 and 8 after aGvHD induction; analysis of BM controls was performed on day 6. *N* = 8 (BM), *N* = 5/5 (GR^flox^/GR^lysM^; day 4), *N* = 20/22 (GR^flox^/GR^lysM^; day 6), *N* = 5/7 (GR^flox^/GR^lysM^; day 8); data pooled from multiple experiments. All values are depicted as the mean ± SEM. Survival curves were compared using the Gehan-Breslow-Wilcoxon test, statistical analyses of clinical scores, body temperature and cytokine levels were performed by Mann-Whitney *U* test (^*^p < 0.05; ^**^p < 0.01; ^***^p < 0.001; n.s.: non-significant).

Pro-inflammatory cytokines such as IL-6 are expressed by a variety of recipient cell types. Since macrophages are one of their main sources, we determined the role of the GR in myeloid cells for disease severity and mortality of mice undergoing allogeneic HSCT. To address this issue, we took advantage of GR^lysM^ mice that lack the GR in several myeloid cell types [14] including macrophages that are for the most part resistant to irradiation during aGvHD induction [30]. GR^flox^ control mice died between day 10 and 40 and showed the expected disease course characterized by progressively increasing clinical scores in the early phase of aGvHD and a gradually decreasing body temperature (Figure 3A–3C). In comparison, GR^lysM^ mice developed more severe clinical symptoms on day 6 and 8, which resulted in early lethality before day 10 (Figure 3A, 3B). Furthermore, the body temperature of GR^lysM^ mice on day 8 was strongly reduced compared to GR^flox^ mice, which presumably contributes to their high mortality (Figure 3C). Collectively, GR deletion in recipient myeloid cells largely reproduces the aggravated aGvHD phenotype observed in GR^dim^ mice.

### Disease severity in GR^lysM^ mice does not coincide with target tissue damage

**Figure 4:**
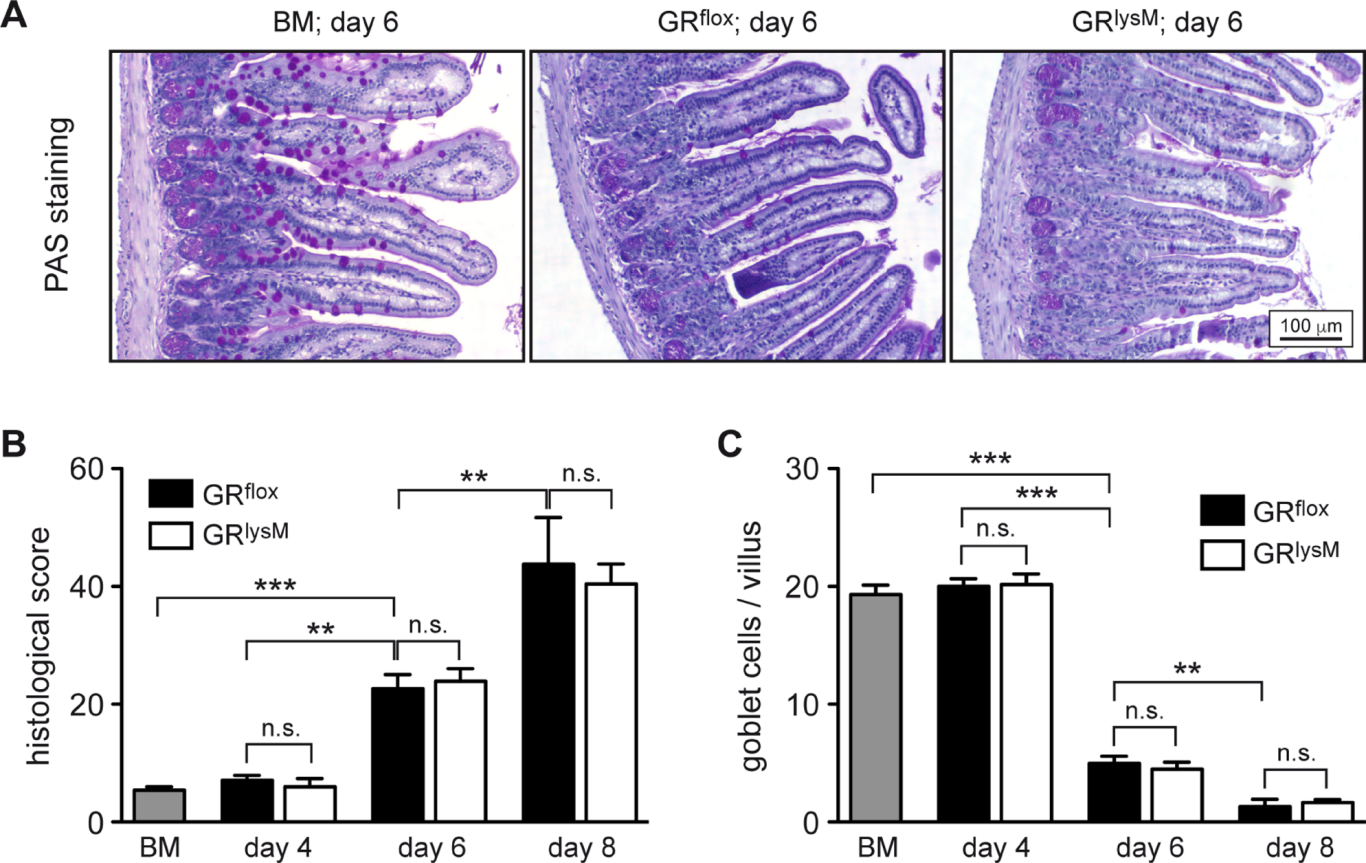
Histological assessment of the jejunum in the early phase of aGvHD in the GR^lysM^ model GR^flox^ and GR^lysM^ BALB/c mice were transplanted with BM and purified T cells from C57BL/6 wildtype mice; transfer of BM cells only served as a control. Mice were sacrificed and analyzed on day 4, 6 and 8 after aGvHD induction; analysis of BM controls was performed on day 6. (**A**) Representative microphotographs of sections of the jejunum collected on day 6 from BM control and aGvHD mice stained by PAS reaction. Size bar: 100 µm. (**B**) Histological scores obtained by assessment of H&E stained jejunum sections from all experimental groups. *N* = 7 (BM), *N* = 5/5 (GR^flox^/GR^lysM^; day 4), *N* = 20/21 (GR^flox^/GR^lysM^; day 6), *N* = 6/7 (GR^flox^/GR^lysM^; day 8); data pooled from multiple experiments. (**C**) Goblet cell numbers per villus determined by PAS staining of jejunum sections from all experimental groups. *N* = 6 (BM), *N* = 5/5 (GR^flox^/GR^lysM^; day 4), *N* = 18/21 (GR^flox^/GR^lysM^; day 6), *N* = 5/8 (GR^flox^/GR^lysM^; day 8); data pooled from multiple experiments. All values are depicted as mean ± SEM. Statistical analyses were performed by Mann-Whitney *U* test (^**^p < 0.01; ^***^p < 0.001; n.s.: non-significant).

**Figure 5:**
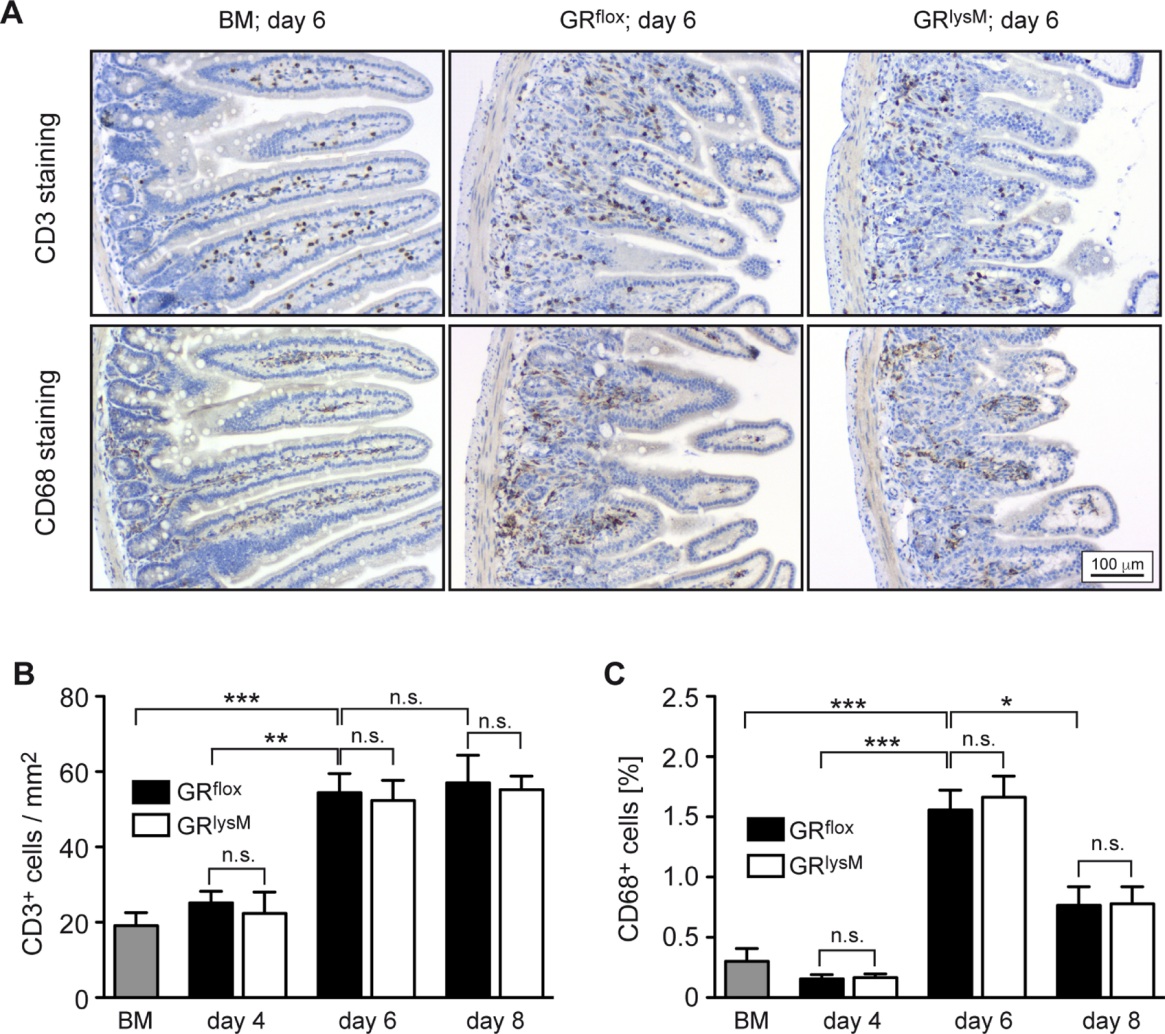
Immunohistochemical assessment of the jejunum in the early phase of aGvHD in the GR^lysM^ model GR^flox^ and GR^lysM^ BALB/c mice were transplanted with BM and purified T cells from C57BL/6 wildtype mice; transfer of BM cells only served as a control. Mice were sacrificed and analyzed on day 4, 6 and 8 after aGvHD induction; analysis of BM controls was performed on day 6. (**A**) Representative microphotographs of sections of the jejunum collected on day 6 from BM control and aGvHD mice and stained with antibodies recognizing CD3 (upper panel) or CD68 (lower panel). Size bar: 100 µm. (**B**) Numbers of CD3^+^ cells per mm^2^ were determined by computer-aided counting of stained cells in jejunum sections from all experimental groups using ImageJ software. *N* = 7 (BM), *N* = 5/5 (GR^flox^/GR^lysM^; day 4), *N* = 20/21 (GR^flox^/GR^lysM^; day 6), *N* = 6/7 (GR^flox^/GR^lysM^; day 8); data pooled from multiple experiments. (**C**) CD68^+^ cells were enumerated in jejunum sections from all experimental groups by measuring the percentage of stained area using ImageJ software. *N* = 6 (BM), *N* = 5/5 (GR^flox^/GR^lysM^; day 4), *N* = 20/20 (GR^flox^/GR^lysM^; day 6), *N* = 6/7 (GR^flox^/GR^lysM^; day 8); data pooled from multiple experiments. All values are depicted as mean ± SEM. Statistical analyses were performed by Mann-Whitney *U* test (^*^p < 0.05; ^**^p < 0.01; ^***^p < 0.001; n.s.: non-significant).

To identify the pathogenic mechanisms explaining the difference in aGvHD severity between GR^flox^ and GR^lysM^ mice, we performed histological analyses of the intestine, one of the major aGvHD target organs. Histopathological assessment revealed significant tissue damage in the jejunum in the early phase of the disease, which was characterized by inflammatory infiltrates, apoptotic cells, villus blunting, edema, and a massive loss of goblet cells (Figure 4A). Tissue damage strongly increased from day 4 to day 8, but despite the exacerbated disease in GR^lysM^ mice, histological scores and the number of goblet cells per villus were similar in both genotypes at all times (Figure 4B, 4C). In addition, we studied CD3^+^ T cells and CD68^+^ myeloid cells in sections of the jejunum by immunohistochemical staining (Figure 5A). Although the numbers of both types of immune cells were significantly elevated in aGvHD mice on day 6, there were no differences in cellular infiltration between GR^flox^ and GR^lysM^ mice at any of the time points analyzed (Figure 5B, 5C). Remarkably, T cell numbers did not further increase on day 8 and macrophage numbers even decreased, probably reflecting the extensive distortion of the villi at this advanced stage of the disease (Figure 5B, 5C). Taken together, our findings indicate that the increased severity of aGvHD in GR^lysM^ mice neither coincides with leukocyte infiltration nor subsequent tissue damage in the jejunum.

### GR deficiency in recipient myeloid cells has only a minor effect on local cytokine production in the jejunum

**Figure 6:**
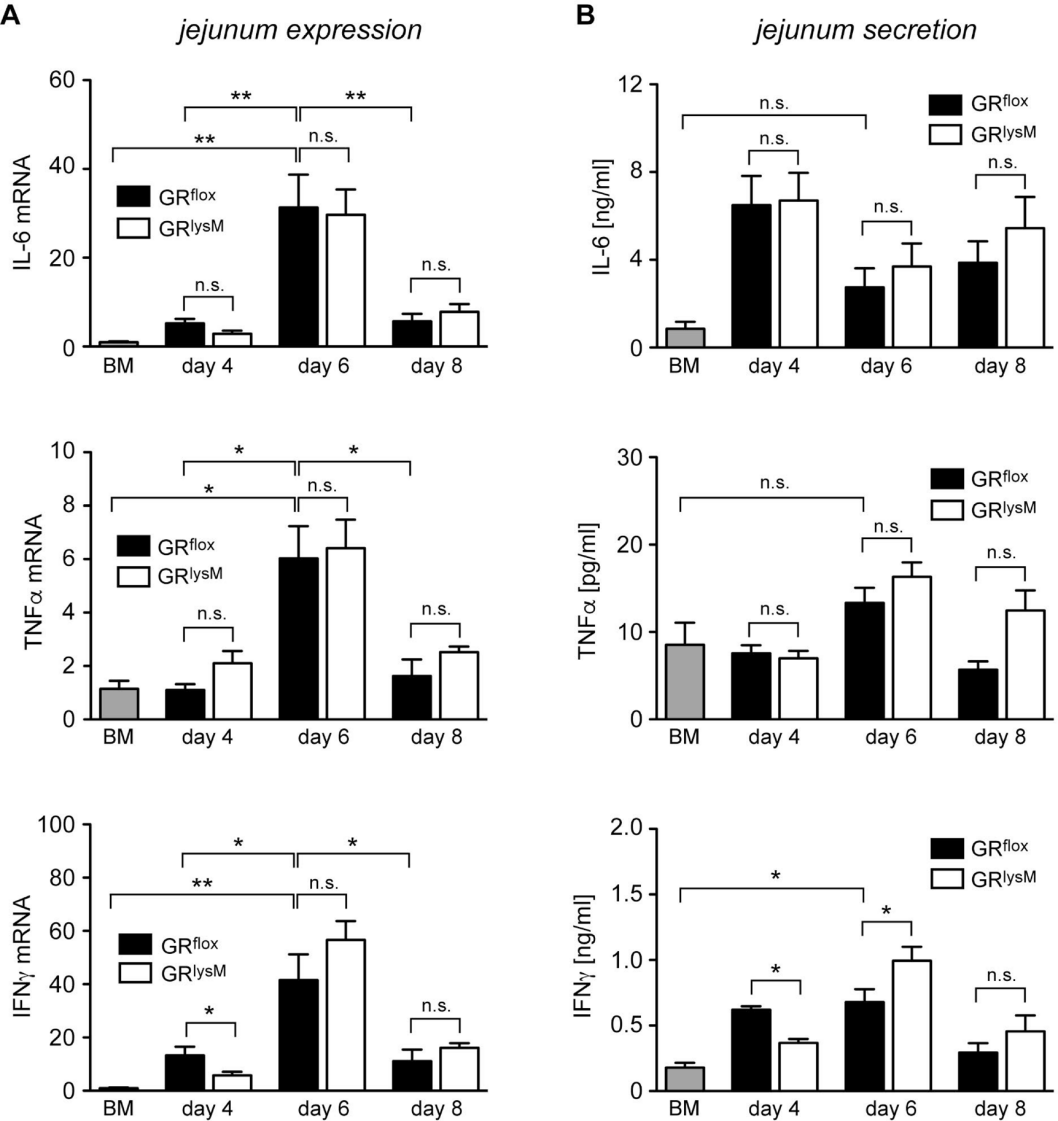
Cytokine expression and secretion in the jejunum in the early phase of aGvHD in the GR^lysM^ model GR^flox^ and GR^lysM^ BALB/c mice were transplanted with BM and purified T cells from C57BL/6 wildtype mice; transfer of BM cells only served as a control. Mice were sacrificed and analyzed on day 4, 6 and 8 after aGvHD induction; analysis of BM controls was performed on day 6. (**A**) Relative mRNA levels of IL-6, TNFα and IFNγ in jejunum biopsies were determined by quantitative RT-PCR using HPRT for normalization. Gene expression in BM control mice was arbitrarily set to 1. *N* = 4 (BM), *N* = 5/5 (GR^flox^/GR^lysM^; day 4), *N* = 12/15 (GR^flox^/GR^lysM^; day 6), *N* = 5/7 (GR^flox^/GR^lysM^; day 8); data pooled from multiple experiments. (**B**) Jejunum biopsies were cultured for 24 hours in RPMI+ medium and IL-6, TNFα and IFNγ levels in the supernatant were determined by ELISA. *N* = 4 (BM), *N* = 5/5 (GR^flox^/GR^lysM^; day 4), *N* = 14/15 (GR^flox^/GR^lysM^; day 6), *N* = 6/8 (GR^flox^/GR^lysM^; day 8); data pooled from multiple experiments. All values are depicted as mean ± SEM. Statistical analyses were performed by Mann-Whitney *U* test (^*^p < 0.05; n.s.: ^**^p < 0.01; n.s.: non-significant).

A major function of the GR in macrophages is the suppression of pro-inflammatory mediators [31]. Hence we started to investigate whether GR ablation in myeloid cells had any impact on local cytokine production in aGvHD target organs. Initially, we isolated RNA from the jejunum and analyzed it by quantitative RT-PCR. IL-6, TNFα and IFNγ mRNA levels strongly increased from day 4 to day 6 and declined again on day 8. IL-6 and TNFα expression in GR^flox^ and GR^lysM^ mice was always similar while IFNγ was slightly reduced on day 4 (Figure 6A). Of note, a similar pattern of gene regulation was observed in the liver, another major aGvHD target organ (data not shown). To obtain insights into the local release of cytokines, we cultured jejunum biopsies for 24 hours and determined cytokine levels in the supernatant by ELISA. Secretion of IL-6, TNFα and IFNγ in the jejunum did not match the regulatory pattern observed for the respective mRNA levels and showed considerable fluctuations during the early phase of the disease (Figure 6B). There were no differences concerning the release of IL-6 and TNFα between both genotypes whereas the release of IFNγ was slightly diminished on day 4 and elevated on day 6 (Figure 6B). Collectively, these results suggest that GR deficiency in recipient myeloid cells has no major impact on local cytokine production in aGvHD target organs.

### Systemic cytokine secretion in GR^lysM^ mice is strongly increased

**Figure 7:**
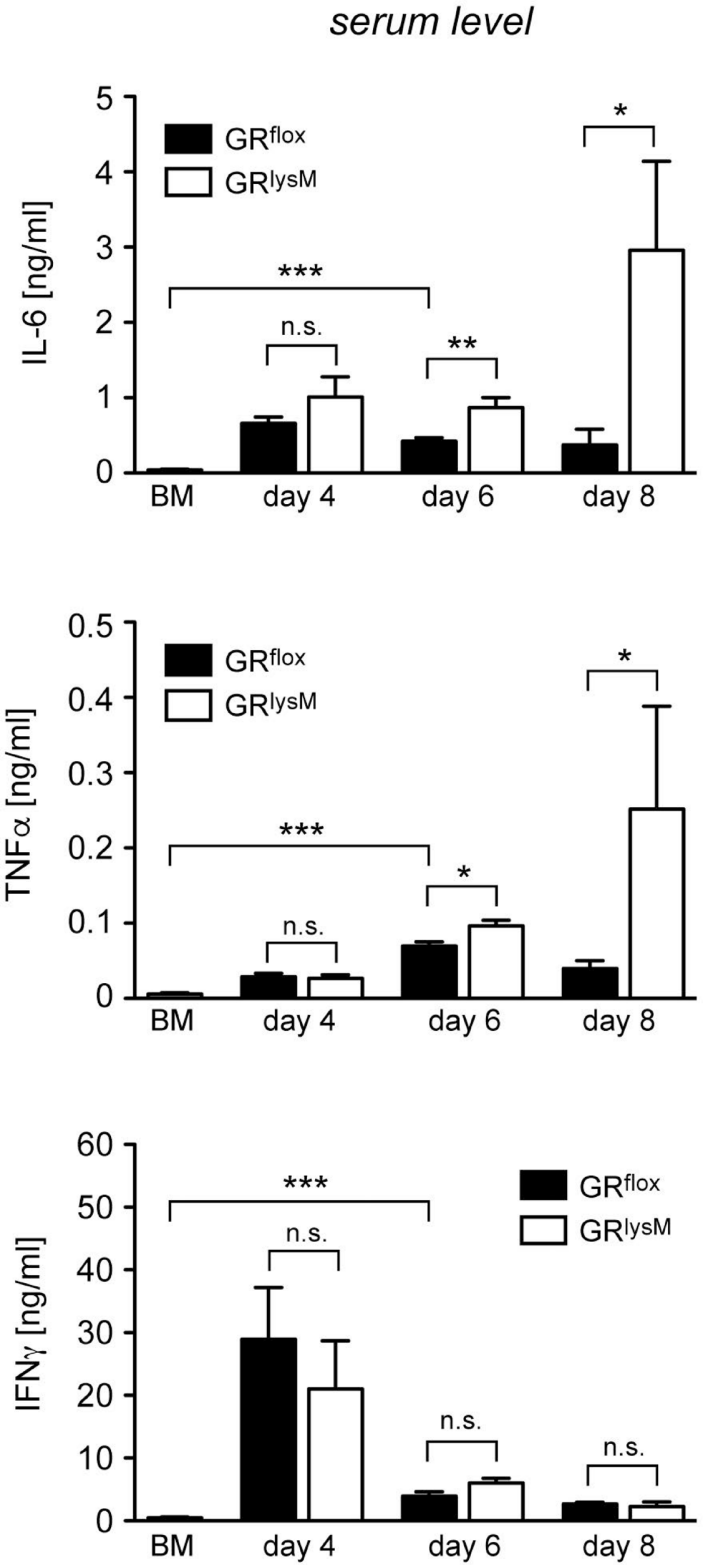
Cytokine serum levels in the early phase of aGvHD in the GR^lysM^ model GR^flox^ and GR^lysM^ BALB/c mice were transplanted with BM and purified T cells from C57BL/6 wildtype mice; transfer of BM cells only served as a control. Mice were sacrificed and analyzed on day 4, 6 and 8 after aGvHD induction; analysis of BM controls was performed on day 6. Serum levels of IL-6, TNFα and IFNγ were determined by ELISA and are depicted as mean values ± SEM. *N* = 12 (BM), *N* = 5/5 (GR^flox^/GR^lysM^; day 4), *N* = 27/28 (GR^flox^/GR^lysM^; day 6), *N* = 6/7 (GR^flox^/GR^lysM^; day 8); data pooled from multiple experiments. Statistical analyses were performed by Mann-Whitney *U* test (^*^p < 0.05; ^**^p < 0.01; ^***^p < 0.001; n.s.: non-significant).

Finally, we tested whether the fulminant disease in mutant mice coincided with unleashed systemic cytokine secretion. IL-6 and TNFα serum levels in diseased GR^flox^ mice were strongly increased in comparison to BM controls during the entire early phase of aGvHD (Figure 7). Systemic release of these two cytokine was similar in both genotypes on day 4 while it was modestly higher in GR^lysM^ mice on day 6 (Figure 7). Around the time when the majority of mutant mice succumb to the disease, secretion of IL-6 and TNFα in mutant mice was massively increased. IL-6 serum levels in GR^lysM^ mice on day 8 were eightfold higher than in GR^flox^ mice and TNFα levels were sixfold elevated (Figure 7). In contrast, secretion of IFNγ was highest on day 4 and then declined without being significantly different between both genotypes at any time point analyzed (Figure 7). We conclude that deletion of the GR in recipient myeloid cells results in a cytokine storm characterized by fulminant systemic IL-6 and TNFα release, which progressively exacerbates until most GR^lysM^ mice succumb to the disease.

## DISCUSSION

Different types of hematopoietic and non-hematopoietic cells contribute to the pathogenesis of aGvHD, and all of them express the GR. Our previous study revealed that T cells contained in the transplant are crucial targets of GC since deleting the GR in these cells resulted in an exacerbated disease course and early lethality [24]. It turned out that GC serve to repress pro-inflammatory cytokines and cytotoxic molecules in transplanted T cells and thereby reduce tissue damage. Using a single MHC class I disparate aGvHD mouse model we could further demonstrate that cytotoxic T cells were the predominant cell type that needs to be controlled by GC in order to prevent fulminant disease [24]. However, it remained elusive up to now whether the role of GC was restricted to transplanted T cells or whether their impact on cells of the recipient was also important. Our current data provide evidence that this is indeed the case. While GR expression in recipient myeloid cells had no influence on the extent of tissue damage, suppression of macrophages by endogenous GC was essential to counteract systemic cytokine release in the early phase of the disease.

GR knock-out mice are not viable due to a defect in lung maturation [25]. Hence we took advantage of GR^dim^ mice to investigate the role of GC in recipient cells. These mice harbor a point mutation which disrupts the GR dimerization interface [15]. Consequently, gene regulation by GC is largely abolished, in particular as far as homodimeric DNA-binding of the GR is concerned [26]. Previous studies in several models of sepsis had revealed that GR^dim^ mice are highly prone to systemic inflammation, resulting in an enhanced mortality due to the inability to downregulate pro-inflammatory cytokines [17, 32]. Just like sepsis, aGvHD is also characterized by systemic inflammation. Thus aGvHD induction is accompanied by increased levels of pro-inflammatory cytokines, an effect which was strongly aggravated in GR^dim^ mice. While LPS-induced sepsis in these mutants could be counteracted by application of an IL-1R antagonist [17], the results presented here revealed that anti-IL-6 antibody treatment reduced disease severity and mortality in a mouse model of aGvHD. Collectively, these findings indicate that the GR is required to limit systemic inflammation caused by cytokine hypersecretion.

Having found that suppression of IL-6 release by cells of recipient mice was crucial for the control of aGvHD, we aimed to identify the cell type being responsible for this effect. Cytokines such as IL-6 are produced by a variety of hematopoietic and non-hematopoietic cells. Since macrophages are an important source of these mediators, we studied aGvHD induction in GR^lysM^ mice. Notably, the mutants express a cre recombinase that causes GR deletion in various myeloid cell types such as neutrophils and macrophages [14]. However, amongst those ones only the latter resist irradiation during aGvHD induction [30]. Analysis of disease symptoms and mortality revealed that aGvHD was strongly aggravated in GR^lysM^ mice compared to GR^flox^ controls, although tissue damage and numbers of T cells and myeloid cells in the gastrointestinal tract, one of the major aGvHD target organs, were surprisingly similar in both genotypes. This finding led us to conclude that repression of myeloid cells by endogenous GC is crucial for aGvHD outcome albeit fulminant disease in the absence of the GR does not coincide with tissue damage in the jejunum in the initial phase of the disease.

IL-6, TNFα and IFNγ are key pathogenic molecules in inflammatory diseases such as aGvHD. Accordingly, blockade of IL-6 ameliorated aGvHD in mice and tocilizumab, a monoclonal antibody that binds to the IL-6 receptor, shows activity in patients that failed to improve after GC administration [28, 29, 33]. Similarly, neutralization of TNFα prolonged survival in a mouse model of aGVHD and administration of the anti-TNFα monoclonal antibody infliximab is associated with an efficient clinical response [34, 35]. In contrast, the exact role of IFNγ in aGvHD remains controversial [36]. Experimental data suggest that IFNγ is not required for the development of aGvHD and may even exert an inhibitory effect [37]. It is against this background that we analyzed regulation of these cytokines in GR^lysM^ mice. GR ablation in myeloid cells had basically no effect on gene expression in the jejunum. IL-6, TNFα and IFNγ mRNA levels increased from day 4 to day 6 regardless of the genotype and declined again on day 8. This expression pattern presumably reflects the inflammatory response that progressively develops in the first few days after HSCT and is followed by a transient resolution [38]. Local cytokine release followed a different pattern that was characterized by considerable fluctuations but again, we hardly observed any differences between mice of both genotypes irrespective of the stage of the disease. In stark contrast to the local regulation of cytokine production, systemic IL-6 and TNFα release was strongly enhanced in mutant mice. While this difference was still moderate on day 6, fulminant cytokine secretion in GR^lysM^ mice was observed around the peak of the disease on day 8 when the majority of mutant mice succumb to the disease [24, 27]. In contrast, IFNγ serum levels in mice of both genotypes were highest on day 4 and declined thereafter, which coincides with the onset of T cell infiltration into the intestinal tract [38]. Taken together, we propose that the fulminant disease in GR^lysM^ mice is a consequence of the overshooting systemic release of IL-6 and TNFα whereas local production of these cytokines in aGvHD target organs is less critical. This situation can presumably be explained by the different origin of myeloid cells in distinct locations in the body. Myeloid cells in non-inflamed organs of GR^lysM^ mice are refractory to the repressive effects of GC due to the ablation of the GR. In contrast, many of the myeloid cells present in aGvHD target organs express the GR since they are derived from wildtype monocytes that are contained in the graft and preferentially migrate to the sites of inflammation. Thus one can expect differences in cytokine regulation to be more pronounced on the systemic than on the local level. Collectively, our findings suggests that the exacerbated cytokine storm is the major cause of mortality in GR^lysM^ mice, which is in accordance with a study in a model of LPS-induced sepsis where increased lethality of mutant mice has also been linked to cytokine hypersecretion [17].

GC are employed to treat inflammatory conditions in the context of allergy, autoimmunity, transplantation, and infectious diseases, but therapy is complicated by side effects that arise due to unspecific activities of these drugs. Accordingly, first-line therapy of aGvHD can cause muscle atrophy, osteoporosis or diabetes [9, 10, 39]. To overcome these limitations, targeted administration of GC would be desirable. Our findings reported here suggest that selective GC delivery to macrophages might allow ameliorating some aGvHD symptoms while causing less side effects. A variety of carrier systems have been developed for GC application during recent years and tested in different models of inflammation and cancer [40]. For example, treatment with Dex palmitate, a liposteroid preferentially taken up by macrophages, attenuated clinical symptoms and improved survival in an aGvHD mouse model, thereby confirming the feasibility of drug targeting in this disorder [41]. Based on these findings and the ones presented here, it is intriguing to speculate that therapies involving the specific delivery of GC to macrophages might result in a better benefit-to-risk profile compared to currently available regimens.

## MATERIALS AND METHODS

### Ethics statement

All animal experiments were conducted according to national and international guidelines and approved by the responsible authority of Lower Saxony (*Niedersächsisches Landesamt für Verbraucherschutz und Lebensmittelsicherheit*).

### Animal experimentation

GR^wt^ and GR^dim^ (Nr3c1^tm3GSc^) mice as well as GR^flox^ and GR^lysM^ (Nr3c1^tm2GSc^Lyz2^tm1(Cre)Ifo^) mice were on a BALB/c background and described earlier [14, 15]. C57BL/6 wildtype mice were purchased from Charles River (Sulzfeld, Germany) or Janvier Labs (St. Berthevin, France). All mice were kept in individually ventilated cages under specific-pathogen-free conditions at our animal facility at the University Medical Center Goettingen, supplied with food and water *ad libitum*, and used at an age of 8–12 weeks. To neutralize IL-6, mice were injected intravenously (i.v.) on day 2, and if applicable, additionally on day 6 with 100 µg of an anti-IL6 antibody (clone MP5-20F3, 1 mg/ml; eBioscience, Frankfurt, Germany).

### aGvHD mouse model

BM was isolated from tibiae, femura and humeri of C57BL/6 wildtype mice and passed through a 100 µm cell strainer. T cells were depleted from the BM by using anti-CD90.2 microbeads (Miltenyi Biotech, Bergisch Gladbach, Germany) or the EasySep™ Positive Selection Mouse CD90.2 Kit II (StemCell Technologies, Grenoble, France). Contaminating T cells in BM preparations were <1% as assessed by flow cytometry. Mature T cells were prepared from spleen and lymph nodes by passing the freshly dissected organs through a 40 µm cell strainer and subsequent purification with the help of the EasySep™ Negative Selection Mouse T Cell Isolation Kit as per the manufacturer’s instructions (StemCell Technologies). Flow cytometric analysis revealed that the purity of T cell preparations was routinely >95%.

BALB/c mice of the different genotypes were subjected to total body irradiation with a dose of 8.5 Gy using an X-Ray source operated at 200 kV, 15 mA and 0.5-mm Cu filtration. On the next day, 1 × 10^7^ BM cells and 2 × 10^6^ T cells were injected via the tail vein to induce aGvHD. Injection of 1 × 10^7^ BM cells only served as a control. Antibiosis was achieved by supplementing the drinking water with 25 µg/ml neomycin for up to three weeks starting one day prior to irradiation. Disease severity was monitored using a scoring system based on five parameters: posture, activity, fur ruffling, diarrhea and weight loss [24]. Each parameter was assigned a score between 0 (no symptoms) and 2 (severe symptoms), resulting in a total score of 0 to 10. Mice which died or had to be sacrificed for ethical reasons were assigned a score of 10 for the rest of the experiment. The body temperature of mice was measured using a BIO-TK9882 thermometer which was equipped with a BIO-BRET-3 rectal probe (Bioseb, Vitrolles, France).

### ELISA

Blood samples were obtained by cardiac puncture, left for coagulation at room temperature for 30 minutes, and centrifuged to separate the serum. The jejunum was flushed with ice-cold PBS and four pieces of 5 mm length from different sites of the jejunum were incubated for 24 hours in RPMI medium supplemented with 10% FCS and 1% Penicillin/Streptomycin (designated RPMI+) at 37° C in a 5% CO_2_ atmosphere. The supernatant was removed for analysis and the jejunum pieces were weighted as a means to correct for differences in their size. Levels of IL-6, TNFα and IFNγ were determined by ELISA using commercially available kits according to the manufacturer’s instructions (BioLegend, Uithoorn, The Netherlands).

### Histology

Tissue biopsies were fixed for 48 hours in 4% PFA (Carl Roth, Karlsruhe, Germany) at room temperature and subsequently dehydrated and embedded in paraffin. 2 µm sections were prepared and stained with hematoxylin and eosin (H&E) or by Periodic acid-Schiff reaction (PAS) according to standard protocols. Photomicrographs were acquired using a Leica Axio Scope A1 microscope (Wetzlar, Germany). Histopathological scores were determined in a blinded manner by evaluating four criteria in ten fields per section [42]: (A) villous blunting/flattening (0 = none, 1 = yes; 20× magnification), (B) number of apoptotic cells (40× magnification), (C) grade of inflammation (0 = none, 1 = mild, 2 = moderate without abscess, 3 = presence of abscess, erosions or ulcer; 20× magnification), (D) edema (0 = none, 1 = yes; 20× magnification). The total histopathological score assigned to each section was calculated as the sum of individual scores obtained for each criterion in all fields. The average number of goblet cells per villus was determined by counting PAS-positive cells in a total of ten villi at 40× magnification.

### Immunohistochemistry

For immunohistochemical stainings, 2 μm tissue sections were incubated in EnVision Flex Target Retrival Solution, Low pH (Dako Agilent Technologies, Santa Clara, CA). This step was followed by incubation with primary antibodies recognizing CD3 (1:2000; Santa Cruz Biotechnology) or CD68 (1:200; Abcam, Cambridge, UK) for 30 minutes at room temperature. Polymeric secondary antibodies coupled to HRP (mmPRESS HRP Polymer Detection Kit; Vector Laboratories, Burlingame, CA) and DAB (Dako Agilent Technologies) were employed to visualize the sites of immunoreactivity. Counterstaining was done with hematoxylin. Photomicrographs were acquired using a Leica Axio Scope A1 microscope and quantified after taking ten pictures per section at 20x magnification. To this end, pictures were processed using ImageJ (https://imagej.nih.gov/ij/) either by counting individual cells (CD3) or by measuring the stained tissue areas (CD68).

### RNA isolation and quantitative RT-PCR

Total RNA was isolated with the RNeasy Mini Kit (Qiagen, Hilden, Germany) and reverse transcribed into cDNA using the iScript Kit (Bio-Rad, Munich, Germany). Quantitative RT-PCR was performed on an ABI 7500 instrument (Applied Biosystems, Darmstadt, Germany) by employing the SYBR mastermix from the same company. Results were normalized to mRNA expression of HPRT and evaluated using the ΔΔCt method. All primers were synthesized by Metabion (Planegg, Germany); the sequences are available upon request.

### Statistical analysis

All data were analyzed using Prism^®^ (GraphPad Software, San Diego, CA). The Mann-Whitney *U* test was used for comparisons of two individual groups, a One-way ANOVA followed by Newman-Keuls test was used to analyze multiple groups, and the Gehan-Breslow-Wilcoxon test was employed for the analysis of Kaplan-Meier survival curves. Levels of significance: ^*^p < 0.05, ^**^p < 0.01, ^***^p < 0.001, n.s.: non-significant.
